# A Phase 2a Randomized Study to Evaluate the Safety and Immunogenicity of the 1790GAHB Generalized Modules for Membrane Antigen Vaccine against *Shigella sonnei* Administered Intramuscularly to Adults from a Shigellosis-Endemic Country

**DOI:** 10.3389/fimmu.2017.01884

**Published:** 2017-12-22

**Authors:** Christina W. Obiero, Augustin G. W. Ndiaye, Antonella Silvia Sciré, Bonface M. Kaunyangi, Elisa Marchetti, Ann M. Gone, Lena Dorothee Schütte, Daniele Riccucci, Joachim Auerbach, Allan Saul, Laura B. Martin, Philip Bejon, Patricia Njuguna, Audino Podda

**Affiliations:** ^1^KEMRI-Wellcome Trust Research Programme, Clinical Research Department, Kilifi, Kenya; ^2^GSK Vaccines Institute for Global Health, Siena, Italy; ^3^GSK Vaccines Clinical Laboratory Sciences, Marburg, Germany; ^4^Clinical Tropical Medicine, Nuffield Department of Medicine, University of Oxford, Headington, United Kingdom; ^5^Department of Public Health, Pwani University, Kilifi, Kenya

**Keywords:** *Shigella sonnei*, 1790GAHB vaccine, generalized modules for membrane antigen, safety, immunogenicity, *Shigella*-endemic settings

## Abstract

Shigellosis is a mild-to-severe diarrheal infection, caused by the genus *Shigella*, and is responsible for significant morbidity and mortality worldwide. We evaluated the safety and immunogenicity of an investigational *Shigella sonnei* vaccine (1790GAHB) based on generalized modules for membrane antigens (GMMA) in Kenya, a *Shigella*-endemic country. This phase 2a, observer-blind, controlled randomized study (NCT02676895) enrolled 74 healthy adults aged 18–45 years, of whom 72 were vaccinated. Participants received, in a 1:1:1 ratio, two vaccinations with the 1790GAHB vaccine at doses of either 1.5/25 μg of O antigen (OAg)/protein (group 1.5/25 μg) or 5.9/100 μg (group 5.9/100 μg) at day (D) 1 and D29, or vaccination with a quadrivalent meningococcal vaccine at D1 and tetanus, diphtheria, and acellular pertussis vaccine at D29 (control group). Solicited and unsolicited adverse events (AEs), serious AEs (SAEs), and AEs of special interest (neutropenia and reactive arthritis) were collected. Anti-*S. sonnei* lipopolysaccharide (LPS) serum immunoglobulin G (IgG) geometric mean concentrations (GMC) were evaluated at D1, D29, and D57 and compared to anti-*S. sonnei* LPS antibody levels in convalescent patients naturally exposed to *S. sonnei*. The percentages of participants with seroresponse were also calculated. The most frequently reported solicited local and systemic AEs across all groups were pain and headache, respectively. Only one case of severe systemic reaction was reported (severe headache after first vaccination in group 5.9/100 μg). Seven and three episodes of neutropenia, assessed as probably or possibly related to vaccination respectively, were reported in the investigational and control groups, respectively. No other SAEs were reported. Despite very high baseline anti-*S. sonnei* LPS serum IgG levels, the 1790GAHB vaccine induced robust antibody responses. At D29, GMC increased 2.10- and 4.43-fold from baseline in groups 1.5/25 and 5.9/100 μg, respectively, whereas no increase was observed in the control group. Antibody titers at D57 were not statistically different from those at D29. Seroresponse was 68% at D29 and 90% at D57 in group 1.5/25 μg, and 96% after each vaccination in group 5.9/100 μg. The 1790GAHB vaccine was well tolerated and highly immunogenic in a population of African adults, regardless of the GMMA OAg/protein content used.

## Introduction

Diarrheal diseases are a leading cause of morbidity and mortality among all age groups, and particularly among young children (1). With 164,000 deaths in 2015, *Shigella* is one of the major causes of overall diarrheal mortality (1), second only to rotavirus (2). Although a decline in mortality due to diarrheal diseases has been observed in the last decade in children less than 5 years of age, yearly deaths still ranged between 499,000 (1) and 525,000 (3) in 2015. Most of these fatalities occurred in Sub-Saharan Africa and Asia, and *Shigella* accounted for approximately 11% of them (1). Additionally, in a recent study conducted in these continents, *Shigella* was identified as a significant cause of moderate-to-severe diarrhea in children (4), and its relevance was reinforced when analyses were repeated using molecular diagnostic tests (5).

Among the four species of the genus *Shigella*, the 15 serotypes of *Shigella flexneri* are mostly isolated in developing countries, while the single serotype of *Shigella sonnei* was traditionally encountered in high-income settings. However, this serotype has emerged lately as one of the dominant species also in many regions of Asia, Latin America, and the Middle East (4, 6–8).

An additional element of concern about shigellosis is the decreased susceptibility to a large range of antibiotics observed over the last decades, with most of the *Shigella* serotypes becoming multi-drug resistant (9). This reinforces the need for a widely available vaccine against shigellosis.

Several candidate vaccines, developed using different technologies, are currently under investigation (10). Inactivated vaccine candidates based on O Antigen (OAg), which is recognized as a key target antigen for *Shigella*, including conjugates, bioconjugates, and live-attenuated vaccine strains have already been tested in clinical trials (10–14). Recently, generalized modules for membrane antigens (GMMA) have been proposed as a delivery system for *S. sonnei* OAg (15). GMMA are optimally sized for immune stimulation and have self-adjuvanting activity, delivering innate signals through toll-like receptor ligands and other pathogen-associated molecular patterns. Although alum is not needed as an adjuvant, the vaccine has been formulated with Alhydrogel, which was shown to reduce the pyrogenicity in rabbits (15).

This *S. sonnei* GMMA vaccine has been shown to be highly immunogenic and well tolerated in phase 1 clinical trials, when administered by intramuscular route to healthy European adults (16).

The current study aimed to further evaluate the safety and immunogenicity profile of the 1790GAHB vaccine in healthy adults from Coastal Kenya, a *Shigella*-endemic setting, and assessed two different GMMA OAg/protein doses. As no serologic correlates of protection are established for *S. sonnei* and as the presence of anti-*Shigella* lipopolysaccharide (LPS) antibodies was previously associated with acquired immunity to the pathogen (17), vaccine-induced immunogenicity was compared to anti-*S. sonnei* LPS antibody levels in a naturally infected, convalescent population.

**Figure 1:**
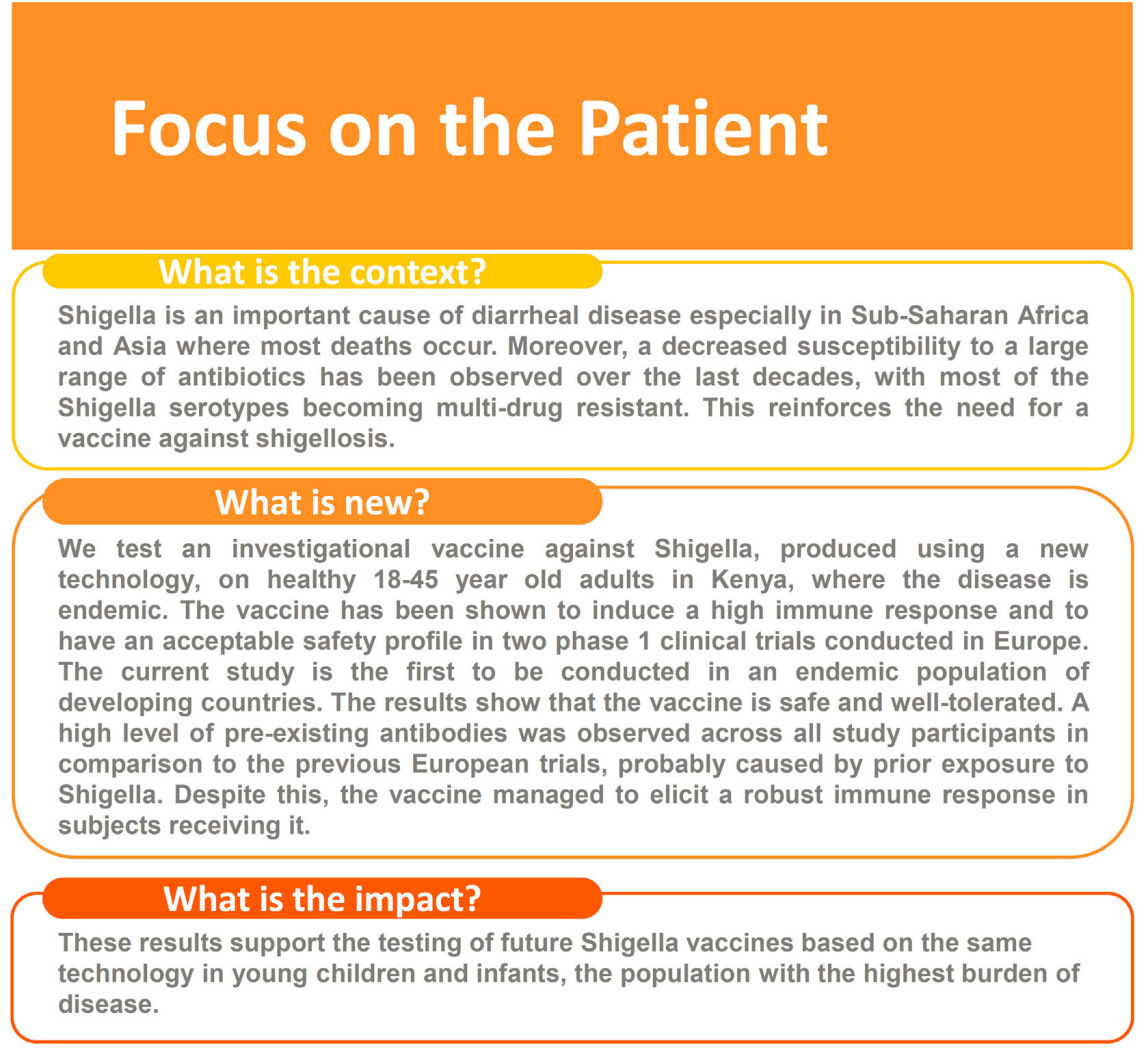
Focus on patient section.

A summary contextualizing the results and potential clinical research relevance and impact is displayed in the Focus on Patient Section (Figure 1) for the benefit of Health Care Professionals.

## Materials and Methods

### Study Design and Participants

This phase 2a, observer-blind, randomized, single-center, controlled study was conducted at the KEMRI-Wellcome Trust in Kilifi, Kenya, between August 2016 and March 2017. The study enrolled healthy adults aged 18–45 years, fulfilling protocol inclusion and exclusion criteria, willing to comply with study procedures and signing, or thumb printing, the informed consent form for study participation. Females of child bearing potential were enrolled only if they agreed to use an effective birth-control method prior to and during the study. However, any potential pregnancies during the trial were to be reported and, if possible, their outcome was to be monitored. Any condition potentially interfering with the ability to participate in the study or with the study results, or causing additional risk by participation in the trial was an exclusion criterion. Individuals were also excluded if they had any of the following conditions: progressive or severe neurological disorders, seizures, previous Guillain–Barré syndrome, history of reactive arthritis, hepatitis B infection, HIV or HIV-related disease, autoimmune disorders, known or suspected impairment/alteration of the immune system, known bleeding diathesis (or any condition that may be associated with prolonged bleeding time), serious chronic or progressive disease, malignancy or lympho-proliferative disorder, history of allergy to vaccine components or of substance or alcohol abuse within the past 2 years, participation in clinical trials with other investigational product within 28 days prior to screening, receipt of vaccines containing meningococcus A, C, W, Y, tetanus, diphtheria or pertussis antigens within 12 months before screening, receipt of any other vaccine within 4 weeks prior to screening or plan to do so during the study, receipt of blood/plasma products within 12 weeks prior to first study vaccination, body mass index >30 kg/m^2^, laboratory confirmed case of disease by *S. sonnei*, and breast-feeding.

Individuals with an absolute neutrophil count (ANC) <1.8 × 10^9^/L for the initial 18 participants (first study cohort) or <1.0 × 10^9^/L, if recommended by an independent Data Safety Monitoring Board (DSMB) for the remaining study population (second cohort) at screening or with previous history of benign ethnic neutropenia/drug-related neutropenia, prior use or likelihood to use neutropenic drugs were not enrolled. Inclusion criteria were re-assessed for all participants, prior to each study vaccination.

**Figure 2:**
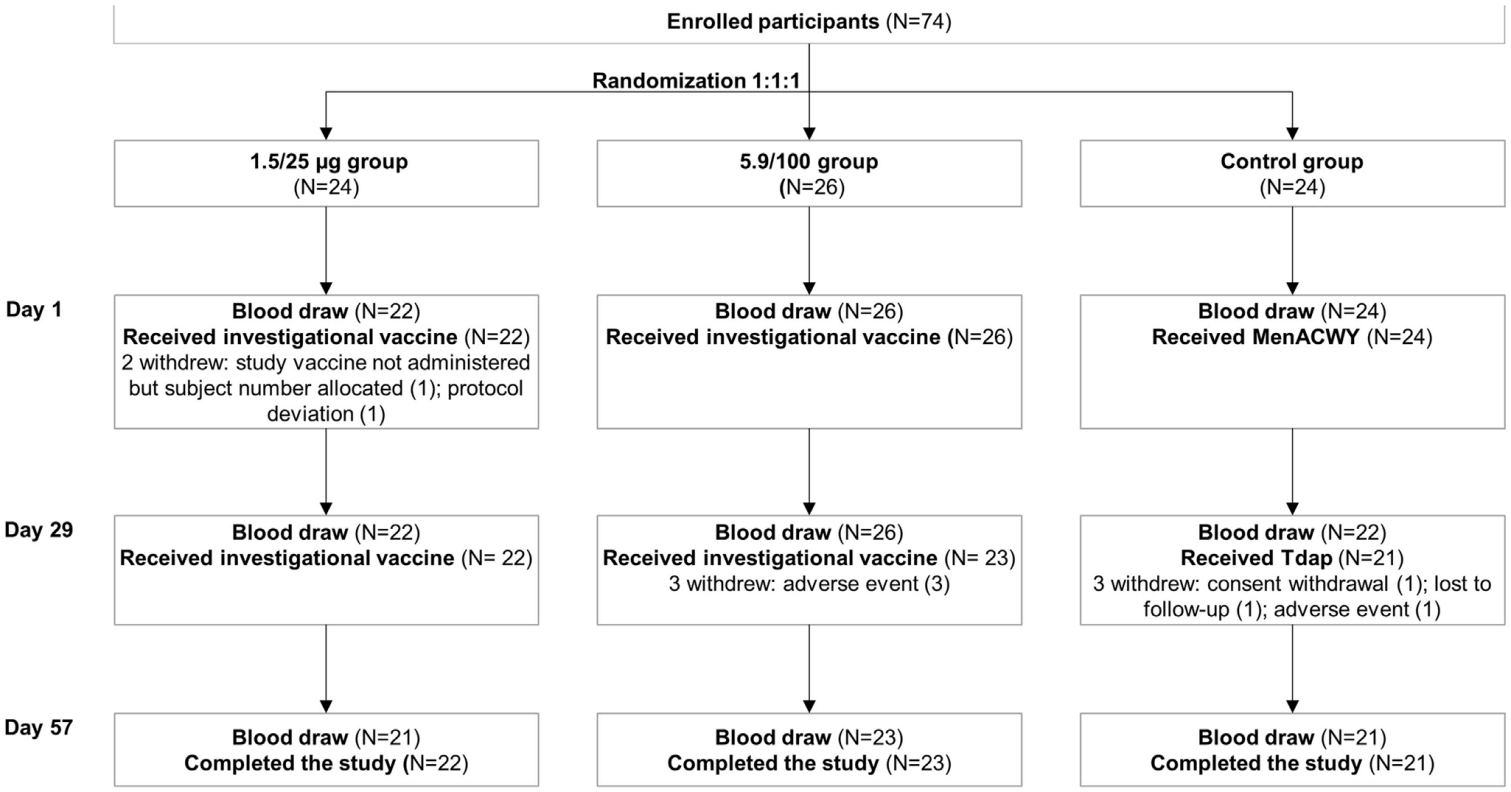
Participant flowchart and timing of blood draws for immunogenicity analyses. *N*, number of participants; 1.5/25 μg, 5.9/100 μg monovalent *Shigella sonnei* 1790GAHB vaccine, with an O antigen/protein content of 1.5/25 and 5.9/100 μg, respectively; MenACWY, meningococcal vaccine against serogroups A, C, W, and Y; Tdap, tetanus, diphtheria, and acellular pertussis vaccine.

Enrolled individuals were randomized in a 1:1:1 ratio, to receive two vaccinations with either the investigational or the control vaccines at day (D) 1 and D29. Investigational groups received the *S. sonnei* 1790GAHB vaccine, used at two different OAg/protein doses, while participants in the control group were randomized to receive a quadrivalent meningococcal conjugate vaccine (MenACWY; *Menveo*, GSK) at D1 and a vaccine against tetanus, diphtheria, and acellular pertussis (Tdap; *Boostrix*, GSK) at D29 (Figure 2).

Randomization was performed by a validated internet-based system. The study was observer-blind, due to the different presentations of the investigational and control vaccines. Designated unblinded trained and qualified staff prepared or administered the study vaccines, but was not involved in the evaluation of the participants for safety or in the collection of study data.

The study was monitored by the DSMB. Eighteen participants were initially enrolled in the first study cohort, received first vaccination and were followed up, according to study procedures, for 7 days. A summary of all safety data and listings including hematology, blood chemistry and urine dipstick/urinalysis test values were provided to the DSMB. After reviewing all safety data, the DSMB recommended that enrollment could be completed using the published African consensus ANC threshold of 1.0 × 10^9^/L (18) as inclusion criterion and the severity grading system proposed by the Division of Acquired Immunodeficiency Syndrome (19), which takes into consideration also the ethnic differences in ANC, be used for classification of postvaccination neutropenia. The DSMB was also consulted for any potential safety issue reported during the trial.

The informed consent form and the study protocol were reviewed and approved by the KEMRI Scientific and Ethics Committee, the Kenyan Pharmacy and Poisons Board, and the Oxford Tropical Research Ethics Committee prior to study start. The trial was designed and conducted in agreement with the ICH Harmonized Tripartite Guidelines for Good Clinical Practice, applicable local regulations and the Declaration of Helsinki and was registered at ClinicalTrials.gov (NCT02676895).

### Study Objectives

The primary objective was to evaluate the safety profile of two vaccinations in healthy adults with two different dose levels of 1790GAHB in a *Shigella*-endemic country. The secondary objective was to assess the immunogenicity of the investigational vaccine, as measured by anti-*S. sonnei* LPS serum immunoglobulin G (IgG) levels, at 28 days after each vaccination.

### Study Vaccines

The 1790GAHB vaccine consisted of *S. sonnei* 1790-GMMA (approximately 11.8 µg OAg/200 μg total protein per milliliter) adsorbed to Alhydrogel (0.7 mg Al^3+^/mL) in tris-buffered saline, was available as a liquid formulation in single-dose vials with 0.7 mL of injectable suspension and did not contain any preservative. A 0.5-mL dose containing 1.5/25 μg of OAg/protein was obtained by bed-side mixing, by dilution with Alhydrogel in tris-buffered saline (0.7 mg Al^3+^/mL).

Each 0.5 mL dose of MenACWY contained 10 µg of serogroup A oligosaccharide and 5 µg of each of serogroups C, W, and Y oligosaccharides conjugated to 32.7–64.1 µg of CRM_197_.

Each 0.5 mL dose of Tdap contained 2.5 Lf diphtheria toxoid, 5 Lf tetanus toxoid, 8 µg pertussis toxoid, 8 µg filamentous hemagglutinin, 2.5 µg pertactin, and 0.5 mg aluminum hydroxide.

At each vaccination, a 0.5-mL vaccine dose was administered intramuscularly into the deltoid area of the non-dominant arm.

### Safety Assessments

Participants were observed at 30 and 60 min after each vaccination for any adverse event (AE). Local (injection site pain, erythema, and induration) and systemic (arthralgia, chills, fatigue, headache, malaise, myalgia, and fever) solicited AEs were recorded on diary cards by study personnel performing daily home visits for 7 days following each vaccination. Unsolicited AEs occurring within 28 days following vaccination were reported by the participants and documented by the investigator, during follow-up clinic visits carried out at 7 and 28 days after each vaccination or in the course of unscheduled visits. Solicited AEs continuing beyond the 7-day period following each vaccination were reported as unsolicited AEs.

All AEs were graded for severity by the investigator. Erythema, induration, and swelling of 25–50 mm, 51–100 mm, and >100 mm and fever as axillary temperatures of ≥38.0–38.9, ≥39.0–39.9, and ≥40.0°C were graded as mild, moderate, and severe, respectively. All other local and systemic AEs, if present, were classified as: mild (present but not interfering with activity), moderate (interfering with activity), and severe (preventing daily activity).

Serious AEs (SAEs), AEs of special interest (AESIs), and AEs leading to withdrawal from the study were collected for the entire duration of the study.

The relationship between study vaccination and unsolicited AEs, medically attended AEs, any new onset of chronic disease, AEs leading to withdrawal, and SAEs were also assessed by the investigator. As defined in the clinical protocol, reactive arthritis and neutropenia were AESIs (the former being a general concern for enteric pathogen vaccinations and the latter due to occurrence of similar events during the phase 1 trials evaluating the 1790GAHB vaccine) and, if present, were to be reported as SAEs.

Blood samples for hematology (white blood cells, red blood cells, hemoglobin, hematocrit, platelets, eosinophils, basophils, neutrophils, monocytes, and lymphocytes) and clinical chemistry (total bilirubin, aspartic aminotransferase, alanine aminotransferase, γ-glutamyl transferase, lactic dehydrogenase, alkaline phosphatase, glucose, blood urea nitrogen, creatinine, sodium, and potassium) testing, and urine dipstick samples (for the assessment of glucose, proteins, pH, ketones, nitrites, and blood levels) were collected at 7 and 28 days post-each vaccination. Urinalysis (white blood cells, red blood cells, casts, and bacteria) was performed if urine dipstick showed deviations from normal values. Laboratory measurements were assessed by the investigators and any abnormality considered as clinically significant was reported as an AE.

All study participants with a neutropenia (ANC < 1.8 × 10^9^/L for adults in the first cohort and ANC < 1.0 × 10^9^/L for individuals from the second cohort), occurring at 7 days after each vaccination, had additional blood draws for repeated complete blood count on a weekly basis until resolution of neutropenia. Occurrence of ANC < 0.5 × 10^9^/L, after the first vaccination, was an exclusion criterion for second vaccination.

### Immunogenicity Assessments

Blood samples for immunogenicity assessments were collected prior to first vaccination and 28 days post-each vaccination (Figure 2). The sera were kept frozen at −80°C at the KEMRI-Wellcome Trust laboratory until shipment to the GSK Clinical Laboratory Sciences (Marburg, Germany), for serologic testing; for each participant, one aliquot of serum was stored in the clinical site laboratory and one aliquot was used for immunogenicity analyses.

Anti-*S. sonnei* LPS serum IgG was measured by an enzyme-linked immunosorbent assay (ELISA), as previously described (15).

Seroresponse to vaccination was defined as an increase in the anti-*S. sonnei* LPS serum IgG level of at least 50% for participants with pre-vaccination levels >50 ELISA units (EU) or an increase of at least 25 EU for participants with pre-vaccination levels ≤50 EU.

In the absence of a correlate of protection, the median anti-*S. sonnei* LPS serum IgG following vaccination was compared to the median level in convalescent patient sera from individuals infected with *S. sonnei*, as previously reported (20). Postvaccination levels of 121 EU were estimated to correspond to the median end point titer of 1:800 measured for the convalescent sera with an ELISA method by Cohen et al. (16).

### Statistical Analyses

A total of 72 participants were planned to be enrolled in the study. No formal statistical sample size was calculated, due to the descriptive nature of the study objectives.

Safety analyses were carried out in all participants from the exposed full analysis set who received at least one study vaccination and had safety data. The number and percentage of participants with AEs, SAEs, AESIs, and deviations from normal ranges of safety laboratory data after vaccination was calculated.

Serologic analyses were performed on participants from the full analysis set who had available ELISA data. The ELISA antibody concentrations were logarithmically transformed (base 10). For each group, geometric mean concentrations (GMC) and their 95% confidence intervals (CIs) were computed by exponentiating (base 10) the mean and 95% CIs of the log10 ELISA concentration. ELISA concentrations below the limit of detection were set to half that limit for the purposes of analysis.

The number and percentage of participants with seroresponse and with postvaccination levels ≥121 EU for anti-*S. sonnei* LPS serum IgG at 28 days post-each vaccination was calculated together with 95% Clopper–Pearson CIs.

Additionally, geometric mean ratios (GMR) were computed for GMC at 1 month after first and second vaccination versus baseline levels (D1). The GMR and 95% CIs were constructed by exponentiating the mean within-subject differences in log-transformed titers and the corresponding 95% CIs.

## Results

### Demographics

A total of 152 adults were screened, 74 were enrolled and randomized, 72 received at least one study vaccination, and 66 completed the study. Main reasons for the 78 screening failures were: not fulfillment of inclusion/exclusion criteria (*n* = 52) and lack of interest to further trial participation, despite being eligible (*n* = 15). Of the two individuals who were not vaccinated after randomization, one declined vaccination and the other was erroneously randomized, after expiry of the allowed 28-day window for screening. The primary reasons for study discontinuation were AEs (*n* = 4), administrative reason (*n* = 1), lost to follow-up (*n* = 1), protocol deviation (*n* = 1), and consent withdrawal (*n* = 1) (Figure 2). The overwhelming majority of the participants were male (88–91% in each group) and of Black origin (≥95% across all groups). Baseline characteristics were well-matched across all vaccine groups (Table 1).

**Table 1:** Baseline characteristics of vaccinated study participants (full analysis set).

Group	1.5/25 μg (*N* = 22)	5.9/100 μg (*N* = 26)	Control (*N* = 24)
Age (mean ± SD), years	24.6 ± 5.81	26.9 ± 8.44	28.3 ± 8.23
Male, *n* (%)	20 (91)	23 (88)	21 (88)
Weight (mean ± SD), kg	59.6 ± 9.02	63.7 ± 9.29	60.4 ± 10.97
Height (mean ± SD), cm	169.7 ± 6.15	169.2 ± 6.73	167.2 ± 7.3
Race, *n* (%)			
Black	21 (95)	26 (100)	24 (100)
White	1 (5)	0	0
BMI (mean ± SD), kg/m^2^	20.7 ± 3.12	22.3 ± 3.34	21.6 ± 3.12

*N, number of exposed participants in each group; n (%), number (percentage) of participants in each category; BMI, body mass index; 1.5/25 μg, 5.9/100 μg, participants who received the 1790GAHB vaccine with an O antigen/protein content of 1.5/25 and 5.9/100 μg, respectively, at days 1and 29; Control, participants who received meningococcal vaccine against serogroups A, C, W, and Y at day 1 and tetanus, diphtheria, and acellular pertussis vaccine at day 29*.

### Safety

**Figure 3:**
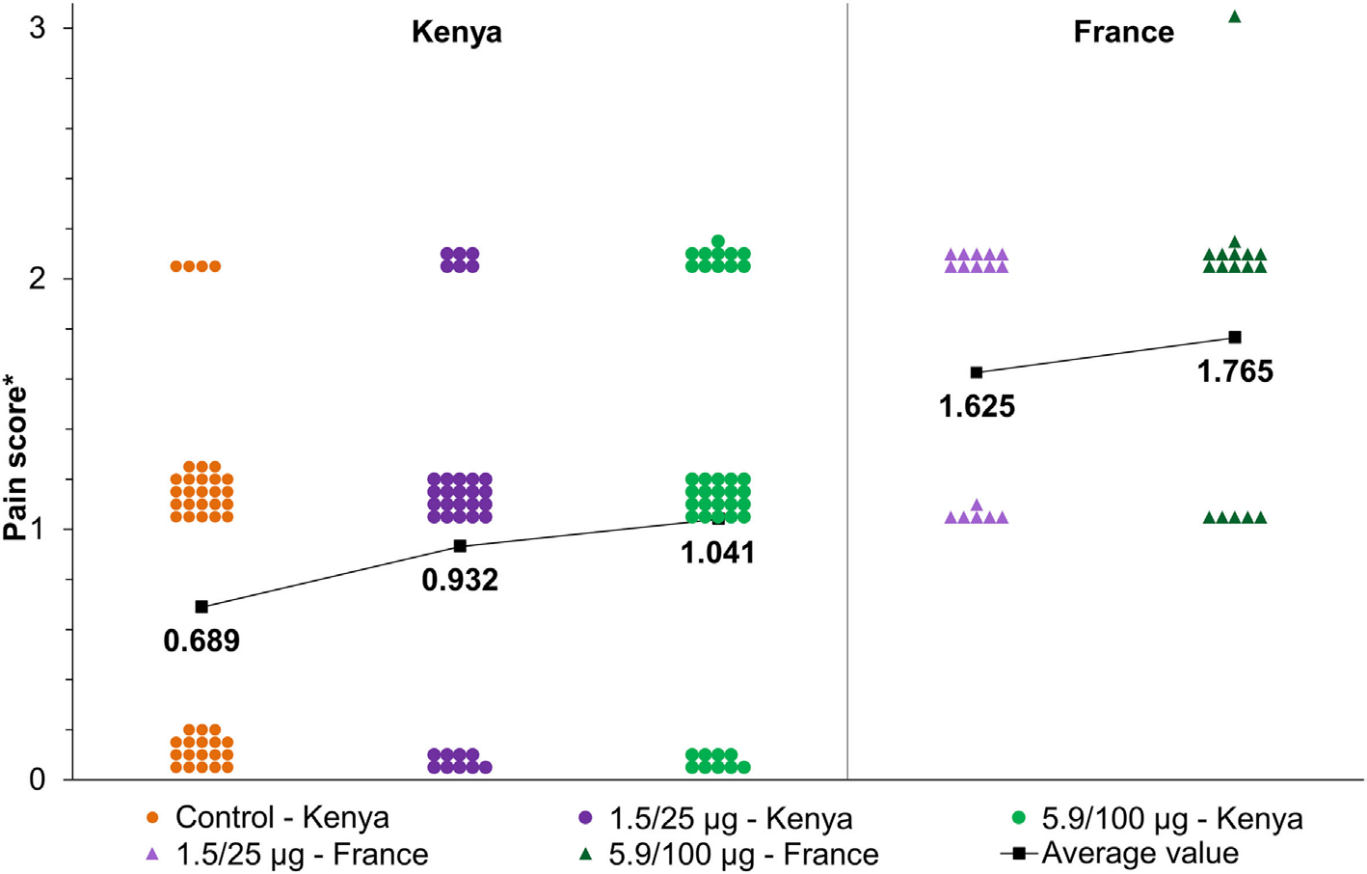
Maximum reported local pain after each vaccination *via* intramuscular route in the current study (NCT02676895) compared to the study conducted in France (NCT02017899) (16). 1.5/25 and 5.9/100 μg, participants who received the 1790GAHB vaccine with an O antigen/protein content of 1.5/25 and 5.9/100 μg, respectively. Note: *Pain score was defined as: 0, no pain; 1, pain present, but does not interfere with activity; 2, pain interferes with activity; 3, pain prevents daily activity. The dots/triangles represent the maximum individual pain reported after each vaccination at different doses. The black line represents the average pain score as a function of dose.

Following the first vaccination, pain was the only reported solicited local AE, in 20 (91%), 25 (96%), and 10 (42%) participants in the 1.5/25 μg, 5.9/100 μg, and control groups, respectively (Table 2). Post-second vaccination, pain was reported by 15 participants in each of the 1.5/25 μg (68%) and 5.9/100 μg (65%) groups, compared with 17 (81%) in the control group, while induration was only reported by 1 (5%) participant in the control group. All reported pain was mild to moderate (Figure 3). No severe local reactions were recorded (Table 2).

**Table 2:** Number and percentage of participants with solicited local and systemic adverse events (AEs) (full analysis set).

Group	First vaccination, *n* (%)			Second vaccination, *n* (%)			Any vaccination, *n* (%)		
	1.5/25 μg (*N* = 22)	5.9/100 μg (*N* = 26)	Control (*N* = 24)	1.5/25 μg (*N* = 22)	5.9/100 μg (*N* = 23)	Control (*N* = 21)	1.5/25 μg (*N* = 22)	5.9/100 μg (*N* = 26)	Control (*N* = 24)
**Solicited local AEs**									

Pain	20 (91)	25 (96)	10 (42)	15 (68)	15 (65)	17 (81)	21 (95)	25 (96)	19 (79)

Severe	0	0	0	0	0	0	0	0	0

Erythema	0	0	0	0	0	0	0	0	0

Severe	0	0	0	0	0	0	0	0	0

Induration	0	0	0	0	0	1 (5)	0	0	1 (4)

Severe	0	0	0	0	0	0	0	0	0

**Solicited systemic AEs**									

Headache	5 (23)	10 (38)	8 (33)	4 (18)	5 (22)	4 (19)	8 (36)	13 (50)	10 (42)

Severe	0	1 (4)	0	0	0	0	0	1 (4)	0

Arthralgia	1 (5)	4 (15)	4 (17)	0	0	1 (5)	1 (5)	4 (15)	5 (21)

Severe	0	0	0	0	0	0	0	0	0

Chills	4 (18)	4 (15)	3 (13)	3 (14)	1 (4)	2 (10)	5 (23)	5 (19)	5 (21)

Severe	0	0	0	0	0	0	0	0	0

Fatigue	2 (9)	8 (31)	7 (29)	2 (9)	1 (4)	4 (19)	3 (14)	9 (35)	9 (38)

Severe	0	0	0	0	0	0	0	0	0

Malaise	3 (14)	3 (12)	6 (25)	2 (9)	0	4 (19)	3 (14)	3 (12)	10 (42)

Severe	0	0	0	0	0	0	0	0	0

Myalgia	0	2 (8)	2 (8)	1 (5)	0	4 (19)	1 (5)	2 (8)	6 (25)

Severe	0	0	0	0	0	0	0	0	0

Fever	1 (5)	1 (4)	0	0	0	0	1 (5)	1 (4)	0

Severe	0	0	0	0	0	0	0	0	0

*N, number of exposed participants in each group; n (%), number (percentage) of participants in each category; 1.5/25 μg, 5.9/100 μg, participants who received the 1790GAHB vaccine with an O antigen/protein content of 1.5/25 and 5.9/100 μg, respectively, at days 1 and 29; Control, participants who received meningococcal vaccine against serogroups A, C, W, and Y at day 1 and tetanus, diphtheria, and acellular pertussis vaccine at day 29*.

The most frequently reported solicited systemic AE was headache, reported by 5 (23%), 10 (38%), and 8 (33%) participants following first vaccination and 4 (18%), 5 (22%), and 4 (19%) participants following the second vaccination, in the 1.5/25 μg, 5.9/100 μg, and control groups, respectively (Table 2). The incidence of all systemic AEs seemed to decrease following the second vaccination and severe reactions were only reported in 1 (4%) participant in the 5.9/100 μg (severe headache occurring 6 h after the first vaccination and only lasting for that day) (Table 2).

Unsolicited AEs following any vaccination were reported by 19 (86%), 24 (92%), and 19 (79%) participants in the 1.5/25 μg, 5.9/100 μg, and control groups, respectively. Possibly or probably related unsolicited AEs were reported by 13 (59%) participants in the 1.5/25 μg and 16 (62%) in the 5.9/100 μg groups, compared to 12 (50%) in the control group. Most of these AEs were post-immunization reactions continuing beyond the 7-day collection window following vaccination (Table 3). Fever was very seldom observed (Table 3) and was never ≥39°C; no participant reported the use of analgesics/antipyretics within 24 h prior to each vaccination.

**Table 3:** Number and percentage of participants with possibly or probably related unsolicited adverse events (AEs) following any vaccination (full analysis set).

Group	1.5/25 μg (*N* = 22)	5.9/100 μg (*N* = 26)	Control (*N* = 24)
Any AE, *n* (%)	13 (59)	16 (62)	12 (50)
Blood and lymphatic system disorders, *n* (%)	2 (9)	3 (12)	1 (4)
Neutropenia	2 (9)	3 (12)	1 (4)
Gastrointestinal disorders, *n* (%)	3 (14)	3 (12)	0
Abdominal pain	0	1 (4)	0
Diarrhea	2 (9)	1 (4)	0
Nausea	1 (5)	1 (4)	0
Vomiting	1 (5)	0	0
General disorders and administration site conditions, *n* (%)	6 (27)	8 (31)	4 (17)
Chills	0	1 (4)	0
Fatigue	1 (5)	2 (8)	1 (4)
Induration	0	1 (4)	0
Injection site pain	6 (27)	7 (27)	1 (4)
Malaise	1 (5)	2 (8)	1 (4)
Pyrexia	0	0	1 (4)
Musculoskeletal and connective tissue disorders, *n* (%)	6 (27)	7 (27)	8 (33)
Arthralgia	0	1 (4)	1 (4)
Limb discomfort	5 (23)	7 (27)	6 (25)
Myalgia	1 (5)	0	2 (8)
Nervous system disorders, *n* (%)	3 (14)	6 (23)	4 (17)
Dizziness	1 (5)	1 (4)	1 (4)
Headache	2 (9)	6 (23)	4 (17)

*N, number of exposed participants in each group; n (%), number (percentage) of participants in each category; 1.5/25 μg, 5.9/100 μg, participants who received the 1790GAHB vaccine with an O antigen/protein content of 1.5/25 and 5.9/100 μg, respectively, at days 1 and 29; Control, participants who received meningococcal vaccine against serogroups A, C, W, and Y at day 1 and tetanus, diphtheria, and acellular pertussis vaccine at day 29*.

During the trial, 10 episodes of neutropenia fulfilling the protocol definition of AESI were reported in two (9%), three (12%), and one (4%) participants in the 1.5/25 μg, 5.9/100 μg, and control groups, respectively. These episodes were all considered probably or possibly related to vaccination, two of them occurred in the 1.5/25 μg group (one mild and one moderate), five in the 5.9/100 μg group (four mild and one moderate), and three in the control group (one mild, one moderate, and one severe). All cases were transient (i.e., recovery within 7 days and by study end) and, except for one participant in the 5.9/100 μg group, who experienced upper respiratory tract infection (D1) and 38.1°C fever (D8), were also asymptomatic, as confirmed by daily home visits during the 7 days postvaccination. Eight of these episodes occurred in 5 of the 18 individuals from the first cohort (screened and monitored using the ANC Western threshold of 1.8 × 10^9^/L), while the remaining two episodes occurred in one of the 54 participants in the second cohort (screened and monitored using the local threshold of 1.0 × 10^9^/L). No SAEs occurred in the study. However, as defined in the study protocol the AESIs were reported as SAEs. No case of reactive arthritis was recorded.

Four participants were prematurely withdrawn due to unsolicited AEs: three in the 5.9/100 μg group (two cases of neutropenia and one case of bone tuberculosis) and one in the control group (γ-glutamyl transferase increase). One participant, who should have been excluded from the second vaccination due to neutropenia, was inadvertently not withdrawn and experienced moderate neutropenia at D57.

Few laboratory abnormalities were considered as clinically significant by the investigators. These were: an increase in alkaline phosphatase levels in one participant in the control group; γ-glutamyl transferase increase in one individual in the control group, and one in the 5.9/100 μg group; decreased hematocrit and hemoglobin levels in one participant in the 5.9/100 μg group; low ANC for one participant in group 5.9/100 μg group; a low platelet count for one participant in the control group; and one increase in platelet count in the 5.9/100 μg group. Following urinalysis, increased leukocyte levels for two, three, and one participants in the 1.5/25 μg, 5.9/100 μg, and control groups, respectively, and high erythrocyte level for one individual in each of the 1.5/25 and 5.9/100 μg groups were considered clinically significant. There were no pregnancies, hospitalization or deaths reported in the study.

### Immunogenicity

**Figure 4:**
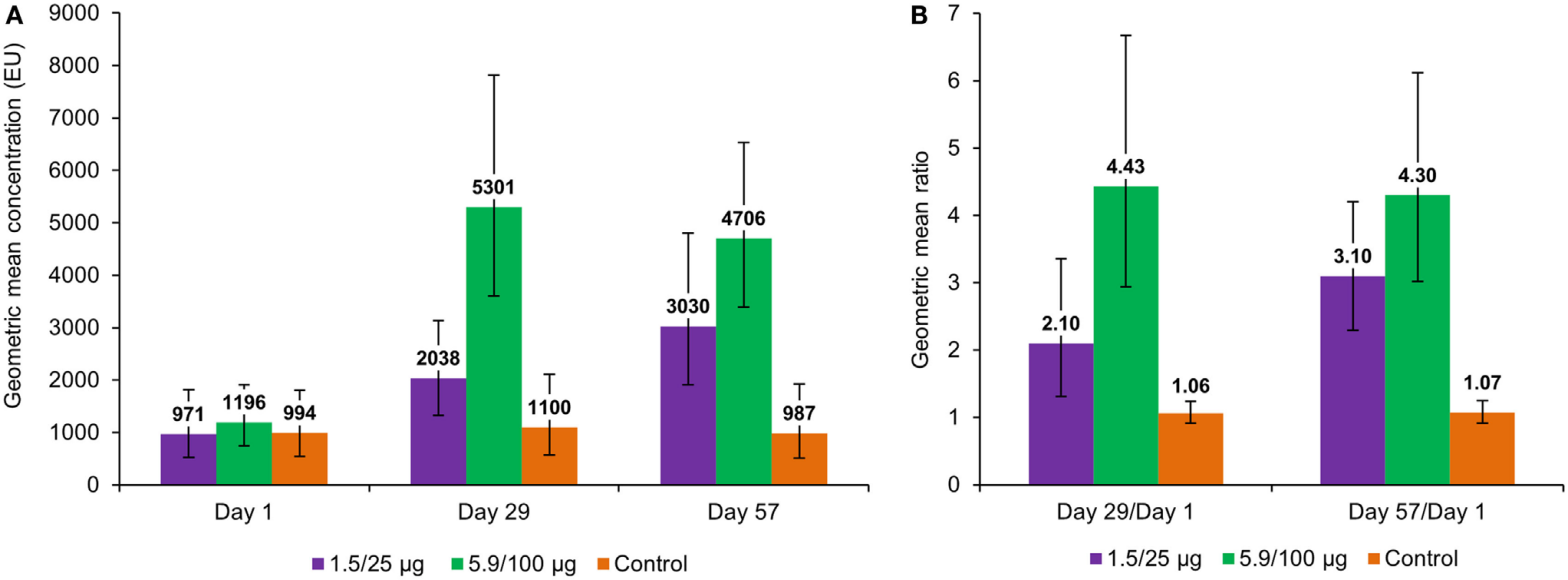
Anti-*Shigella sonnei* LPS IgG geometric mean concentrations **(A)** and geometric mean ratios **(B)**, by timepoint (full analysis set for immunogenicity). LPS, lipopolysaccharide; IgG, immunoglobulin G; EU, enzyme-linked immunosorbent assay units; 1.5/25 μg, 5.9/100 μg, participants who received the 1790GAHB vaccine with an O antigen/protein content of 1.5/25 μg and 5.9/100 μg, respectively, at days 1 and 29; Control, participants who received meningococcal vaccine against serogroups A, C, W, and Y at day 1 and tetanus, diphtheria, and acellular pertussis vaccine at day 29.

Pre-vaccination anti-*S. sonnei* LPS IgG GMC varied between 971 and 1,196 EU across all groups (Figure 4A). At 28 days post-first vaccination, antibody levels increased 2.10- and 4.43-fold from baseline values in the 1.5/25 and 5.9/100 μg groups, respectively, but no increase was observed in the control group (Figures 4A,B). At 28 days after the second vaccination, anti-*S. sonnei* LPS IgG GMC further increased in the 1.5/25 μg group, but not in the 5.9/100 μg group; however, changes observed from D29 to D57 were not statistically significant (Figure 4A).

**Figure 5:**
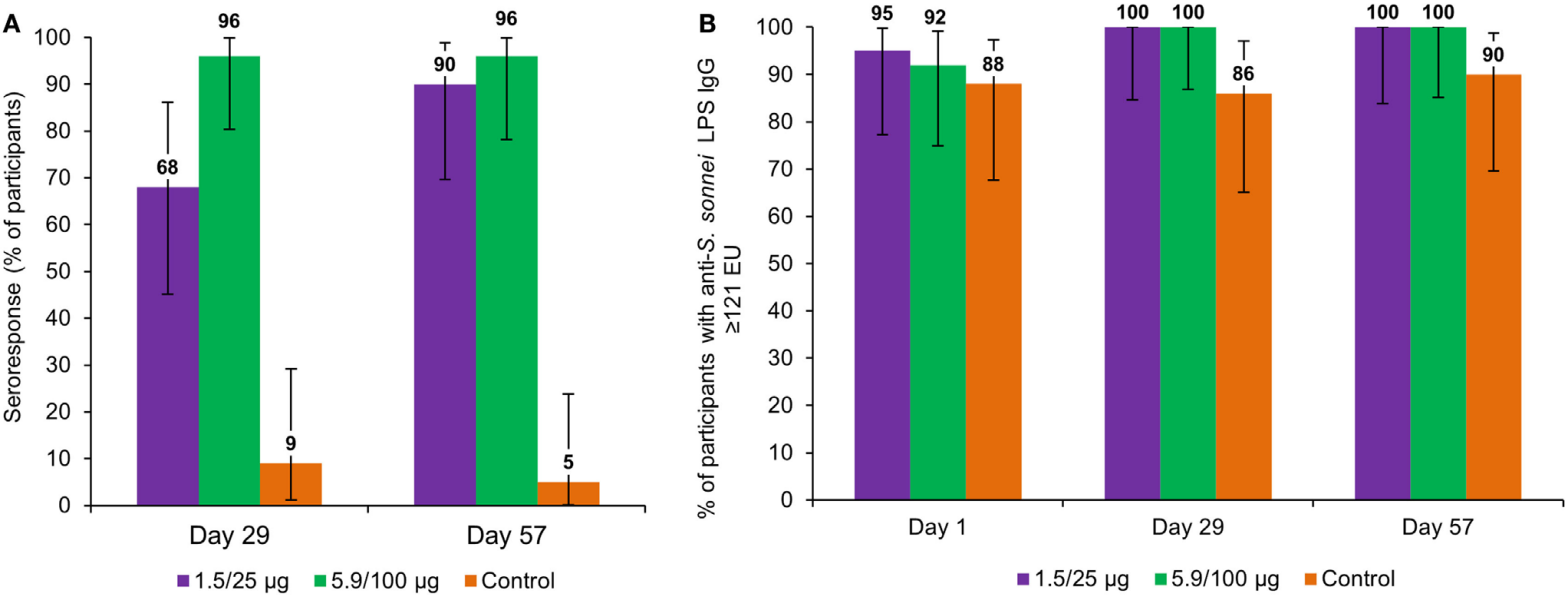
Percentage of participants with seroresponse* **(A)** and anti-*Shigella sonnei* LPS IgG ≥121 EU **(B)**, by timepoint (full analysis set for immunogenicity). LPS, lipopolysaccharide; IgG, immunoglobulin G; EU, enzyme-linked immunosorbent assay units; 1.5/25 μg, 5.9/100 μg, participants who received the 1790GAHB vaccine with an O antigen/protein content of 1.5/25 and 5.9/100 μg, respectively, at days 1 and 29; Control, participants who received meningococcal vaccine against serogroups A, C, W, and Y at day 1 and tetanus, diphtheria, and acellular pertussis vaccine at day 29. Note: *Seroresponse to vaccination was defined as an increase in the anti-*S. sonnei* LPS serum IgG level of ≥50% for participants with baseline levels >50 EU or an increase of ≥25 EU for participants with pre-vaccination levels ≤50 EU.

In the 1.5/25 μg group, the seroresponse was 68% after the first vaccination and 90% after the second vaccination, whereas in the 5.9/100 μg group, seroresponse was 96% after both the first and second vaccination (Figure 5A).

At baseline, the percentages of participants with anti-*S. sonnei* LPS IgG ≥ 121 EU were 95, 92, and 88% for the 1.5/25 μg, 5.9/100 μg, and control group, respectively. At 28 days following each vaccination, all participants in the investigation groups achieved anti-*S. sonnei* LPS IgG ≥ 121 EU, while in the control group 86 and 90% of participants had this level at 28 days post-first and second vaccination, respectively (Figure 5B).

**Figure 6:**
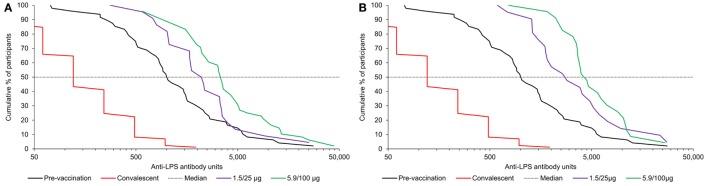
Reverse cumulative distribution curves in participants receiving the 1790GAHB vaccine, following first **(A)** and second **(B)** vaccination compared with baseline and antibody levels in convalescent patient sera from individuals infected with *Shigella sonnei*. LPS, lipopolysaccharide; 1.5/25 μg, 5.9/100 μg, participants who received the 1790GAHB vaccine with an O antigen/protein content of 1.5/25 and 5.9/100 μg, respectively, at days 1 and 29; Convalescent, convalescent patient sera from 87 individuals infected with *S. sonnei* (20).

Reverse cumulative distribution curves for anti-*S. sonnei* LPS antibody levels pre-vaccination and following each vaccination with the 1790GAHB vaccine were compared to antibody levels in convalescent patients as shown in Figure 6. The review of individual immunogenicity results showed that data from five serum samples (out of the 210 collected during the study and shipped for testing), all obtained at D29, were clinically implausible. Most likely, the root cause was human error during sample labeling, executed in the same days for 2 and 3 of the samples, respectively; however, as no definite proof of an error could be established, the original data have been used for the analyses. Additional analyses were performed excluding the potentially invalid results and the interpretation of immunogenicity results did not change.

## Discussion

This is the first study to provide clinical data for a GMMA-based *Shigella* vaccine in a country endemic for shigellosis. For both assessed vaccine strengths, the *S. sonnei* 1790GAHB vaccine was well tolerated; confirming safety results previously shown in age-matched European adults and supporting further potential testing of GMMA-based vaccines in younger individuals from developing countries.

The overall incidence of solicited local and systemic reactions was comparable between the groups receiving the 1790GAHB vaccine and the control vaccines, and very few severe reactions were observed. No increase in the reporting rates was observed following the second vaccination, even in the group receiving the formulation with a higher OAg/protein dose level (5.9/100 μg). Mild to moderate pain at injection site was the only solicited local AE reported in recipients of the investigational vaccine, however, as illustrated in Figure 3, pain intensity appeared to be lower than that previously reported in European age-matched adults (16). The majority of unsolicited AEs were local and systemic reactions continuing beyond the 7-day period following each dose.

Based on prior experience with phase 1 clinical trials (21), neutropenia was followed as an AESI and during this study, 10 episodes occurred in six participants. All but two occurred in the 18 participants from the first cohort, evaluated based on Western ANC normality ranges. Had local ranges been used for the whole study, there would have been only two cases of neutropenia, one mild and one moderate, in one single individual vaccinated with 1790GAHB and one mild episode in one participant from the control group.

These data support previous observations that populations of African descent have a lower ANC than other ethnicities (22, 23) and that in these populations, targeted clinical laboratory reference intervals should be used (18).

The investigational vaccine was highly immunogenic at both assessed OAg/protein contents. Following the first vaccination, a higher increase in anti-*S. sonnei* LPS serum IgG was observed in the 5.9/100 μg group than in the 1.5/25 μg group. Following a second vaccination, anti-*S. sonnei* LPS serum IgG levels further increased in the group receiving the 1.5/25 μg dose, but not in the 5.9/100 μg group.

Of note, a very high level of preexisting antibodies was observed among the study participants, and baseline GMC in all groups were much higher than those from the European study (16) or than median antibody titer established by Cohen et al. in Israeli individuals naturally exposed to *S. sonnei* (20). This finding may be explained by prior and repeated exposure to *S. sonnei* as previously theorized (24, 25). In fact, according to the Global Enteric Multicentre Study, *S. sonnei* is among the predominant *Shigella* species in Kenya (6), and, overall, its prevalence in Africa has increased in the last decades (8). Additionally, *S. sonnei* was recently identified as the main pathogen in young children from Western Kenya hospitals presenting with acute diarrhea between 2011 and 2014, accounting for ~54% of *Shigella* infections (26), so a prior and repeated exposure of the study participants to this pathogen in an hyper endemic country is a strong possibility.

Patients who have a high level of baseline antibody are generally less likely to have further significant increase in antibody levels after immunization, due to masking of the vaccine epitope and/or other mechanisms of specific B cells inhibition (27, 28). By contrast, in our study, we observed a robust specific antibody response in both groups receiving the *Shigella* vaccine, although we also found a reduced fold increase in those subjects with the highest baseline antibodies. Overall the magnitude of the response was much greater than that observed in the European population (16) and this outcome can be considered a strength of the 1790GAHB vaccine. Additionally, compared to vaccines exclusively containing the OAg, GMMA have the advantage of presenting multiple outer membrane antigens to the immune system and induce immunological responses through targets other than OAg. We performed a proteomic analysis of *S. sonnei* GMMA (29) and identified a total of 434 proteins with similar composition and relative abundance to the outer membrane and periplasm of the parent bacteria. The four most abundant proteins by mass were OmpA, OmpC, Entericidin B, and then OmpX.

The lack of information on the potential contribution of protein antigens to the immunological response against *Shigella* is a limitation of this trial and should be further investigated in future studies. In addition, antibody levels were not evaluated beyond 28 days after the second immunization, which does not allow conclusions to be drawn on the persistence of responses to vaccination, and only the quantity but not the quality of antibody response was determined; this latter limitation will be addressed by further testing trial serum samples in a *S. sonnei* serum bactericidal assay. Finally, the sample size of the trial was relatively small and a formal statistical interpretation was not planned.

## Conclusion

The GMMA-based 1790GAHB vaccine against *S. sonnei* displayed good safety and immunogenicity profiles in healthy adults from a shigellosis-endemic country in Africa, thus paving the way for the future testing of multivalent GMMA-based *Shigella* vaccines in young children and infants, the age group with the highest burden of shigellosis in resource-poor populations.

## Ethics Statement

The informed consent form and the study protocol were reviewed and approved by the KEMRI Scientific and Ethics Committee, the Kenyan Pharmacy and Poisons Board and the Oxford Tropical Research Ethics Committee prior to study start. The trial was designed and conducted in agreement with the ICH Harmonized Tripartite Guidelines for Good Clinical Practice, applicable local regulations and the Declaration of Helsinki and was registered at ClinicalTrials.gov (NCT02676895).

## Author Contributions

PN, PB, CO, AS, LM, AP, JA, ASS, and EM were involved in the design of the study. CO, PN, BK, AG, and PB performed the study and participated in the collection or generation of the study data. LS was responsible for generation of the immunogenicity data. All authors were involved in the analyses and interpretation of the data.

## Conflict of Interest Statement

AN, ASS, EM, JA, AS, LM, and AP are all employees of the GSK group of companies and report grants from the EU FP7 (Grants 261472 and 280873) during the conduct of the study and grants from Bill and Melinda Gates Foundation, outside the submitted work. DR and LS are employees of the GSK group of companies. EM, JA, AS, LM, and AP own GSK shares. AS has two patents pending (US2016289632 and US2015202274) and one issued (WO2016202872) to GlaxoSmithKline Biologicals SA. LM has one patent issued (WO2016202872) to GlaxoSmithKline Biologicals SA. CO, PN, BK, AG, and PB declare no conflict of interest. The handling editor declared a past co-authorship with the authors.
